# What Affects the Value of Our Time? The Case of Buying a Present vs. Buying for Ourselves and the Impact of Decision-Making Styles

**DOI:** 10.3390/bs14090786

**Published:** 2024-09-07

**Authors:** Nir Reich, Ofer H. Azar

**Affiliations:** Department of Business Administration, Ben-Gurion University of the Negev, Beer Sheva 84105, Israel; azar@bgu.ac.il

**Keywords:** time valuation, gifts, decision-making styles, consumer behavior, traveling cost

## Abstract

We study how the buying purpose affects the trade-off between time and money. We consider the case of buying something for ourselves versus buying a gift and the decision whether to spend time traveling to a cheaper store to save money. We hypothesized that when purchasing a gift, people make less effort to save money, and therefore will be less willing to spend time traveling to a cheaper store when they buy a gift. In experiments with several scenarios, we fail to find support for this hypothesis. We also explore the impact of telling subjects that the purchase is made abroad. This is hypothesized to increase the valuation of the buyer’s time. We also hypothesize that the interaction of being abroad and buying a gift will be negative. The data support both hypotheses. Subjects answered 15 questions about their decision-making style (rational, intuitive, and spontaneous). Subjects with more rational decision-making exhibit lower time value, which is likely to be closer to the real one. Subjects with more rational decision-making also show more strongly that time abroad is more valuable. These results suggest that questions about decision-making style are correlated with decision-making in scenarios of realistic purchase decisions.

## 1. Introduction

Classical economics assumes that people are rational and make decisions to maximize their utility. However, in recent decades, a new form of theory has developed, aiming to describe how individuals behave in real-life situations, rather than in a “clean” theoretical model that describes how people should behave. One of the important decisions people have to make is how to trade-off time and money (both time and money are valuable resources that are scarce and highly sought. Time, however, unlike money, is available to everyone in equal quantity [1]). This can be relevant when people decide about their labor supply, about how much effort to spend when searching for the lowest price among several sellers, about whether to travel to a distant but cheaper store, and more. Understanding how people trade-off time and money is useful because it can help individuals improve their decision-making and can assist firms, policymakers, and others in responding optimally to the decision-making processes of consumers and citizens.

We add to the literature that examines the trade-off between time and money, analyzing the context of buying something for ourselves versus buying a present for someone else. According to the classical economic theory, the trade-off between time and money (e.g., when we have to decide whether to spend time to go to a cheaper store) should not be affected by whether we purchase something for ourselves or for someone else. It should depend on the value of our time, and the time that can be saved in one option versus the money that can be saved in the other option. We wanted to explore whether this principle holds in actual decisions people make or alternatively whether a bias exists in these decisions.

Moreover, an important perspective is to distinguish between different characteristics and decision-making styles of individuals rather than focusing solely on the decision-making process of the average person. This approach recognizes that different people may approach trade-offs between time and money in varied ways. We distinguish between different decision-making styles: rational, characterized by a thorough search for and logical evaluation of alternatives; intuitive, characterized by a reliance on hunches and feelings; and spontaneous, characterized by a sense of immediacy and a desire to get through the decision-making process as soon as possible [2]. We will analyze how the scores of subjects in the three decision-making styles are related to their decisions in the trade-off between time and money in our scenarios.

Decision-making styles have been found to be relevant for decisions. For instance, personalizing nudges based on people’s decision-making style have been shown to increase security in password creation compared to when personalization was not applied [3], and teams with a more rational decision-making style tend to use more rational strategies [4]. The factorial structure of decision- making styles has been validated in numerous studies, confirming the scale’s reliability and validity [5,6,7,8]. According to the dual-system approach, the rational decision-maker primarily engages System 2, which is associated with deliberate, analytical thinking. In contrast, intuitive and spontaneous decision-makers are more aligned with System 1, which is characterized by fast, automatic, and often emotional processing [9,10].

The importance and contribution of this research lie in understanding the decision-making process regarding the effort individuals invest to save money when purchasing for themselves versus for others, and how different decision-making styles affect decisions in this context.

The literature distinguishes between interpersonal gifting and self-gifting, claiming that people feel less regretful and more cheerful when buying a gift for a friend than for themselves. For instance, hedonic consumption induces less anticipatory guilt in consumers who make purchase decisions for others than in those who make purchase decisions for themselves [11]. People are happier spending money on others than on themselves [12]. When comparing interpersonal gifting versus self-gifting, it was found that people feel more personally rewarded when buying for others than for themselves. Buying for others was significantly more likely to cheer them up, as opposed to buying for themselves. Respondents were significantly more likely to buy for others’ birthdays than they were for their own. Additionally, respondents were more likely to buy gifts for others to be nice to them than they were to buy gifts to be nice to themselves. Individuals were significantly more likely to engage in self-gifting when they had some extra money to spend, whereas buying for others depended on this condition much less [13]. Studying prosocial acts emphasized that what consistently makes people happier is focusing prosocially on others [14].

The considerations when buying a gift also differ based on whether the gift is for oneself or another. People choose differently when deciding for themselves versus for a friend, preferring quality over quantity for friends [15]. One may buy a gift that goes against the buyer’s own identity [16]. Consumers tend to make more indulgent choices for others than for themselves [17].

The allocation of money also depends on whether the purchase is for oneself or others. Consumers manage budgets differently depending on whether the purchases are for personal use or gifts. Given a budget, consumers prefer to minimize expenditures when buying for themselves but to spend close to the budget when buying for others. This behavior is attributed to the less emphasis on savings goals when purchasing gifts [18].

The sensitivity to price differs between self-consumption and social consumption. That is, consumers may have different price sensitivities for the same product category when used in different contexts. For example, when anticipating consuming a product in a social situation, such as eating pizza with others, people tend to purchase more expensive pizzas and are less price-sensitive [19].

Our research extends on the existing literature by studying a topic that was not addressed before in the literature. While prior studies have demonstrated that people experience different emotions and behaviors in the situations of buying for themselves versus buying for others, they did not address the trade-off between time and money in this context. Our study is the first to do so, using scenarios in which the decision-maker can save money by spending time going to a different store. We use the scenarios both for describing purchasing to oneself and for buying a gift for someone else (in a between-subjects design). This allows us to answer for the first time whether people behave differently when they decide how much effort to make to save money in the purchase of a gift versus a purchase for themselves. Moreover, we also study the effect of being abroad (in the scenario) on this trade-off between time and money. Being abroad could be relevant because then the gift recipient is unlikely to discover the gift’s price and is also unable to replace it. In addition, we explore how the type of the decision-maker influences this trade-off between time and money. Since this decision involves non-rational factors, we hypothesize that deviations from rational behavior, specifically the gap in the trade-off between time and money when purchasing for oneself versus for another, will be smaller for rational decision-makers compared to intuitive and spontaneous decision-makers [9,10]. Because decisions about the trade-off between time and money are common in life and because how this trade-off is affected by the buying purpose (buying for ourselves or as a gift) and the decision-making style were not studied before, we believe that our study addresses an important topic, offering original and interesting results.

## 2. Working Hypotheses

The findings that people behave differently when buying for themselves versus for others led us to hypothesize that the trade-off between time and money also depends on the buying purpose: buying a gift for someone else versus buying for oneself. In particular, we consider the case of identifying a specific good that the buyer wants to buy, but having to decide whether to buy the same good at a store currently being visited or at a cheaper store that requires additional effort.

When buying for someone else, an important factor that may affect to what extent the buyer wants to save is the question whether the recipient may try to replace the gift. If he does, the recipient will find out the gift’s price, which can give an incentive to spend more on the gift (compared to a case where the recipient will not try to replace the gift). In addition, spending a larger amount of money on the gift makes the recipient better off when he replaces the gift (having more money to use for the new purchase). We, therefore, hypothesized that the recipient’s ability to replace the gift may affect the willingness to spend time to save money when buying a gift. To gain better insights into this issue, and yet not make the replacement option too emphasized for the subjects, we take advantage of the fact that gifts purchased abroad cannot generally be replaced. Therefore, in one experiment, we consider buying a gift versus buying for oneself (between-subjects) when the purchase is described as being made abroad (with three different questions). In another experiment, the purchase is not abroad, and therefore the subjects may consider the case that the recipient will replace the gift.

We hypothesized that even when the purchase is being made abroad (and therefore the gift cannot be replaced), people will value their time differently and make more effort to save (require a smaller discount for the same effort) when buying for themselves. The reason is that we believe that gifts are measured, at least partially, by the price paid for them. This means that when buying for themselves, subjects may view the entire discount as a pure benefit, whereas when buying a gift for a friend, the savings are partially offset by the negative effect of having purchased a cheaper gift. This hypothesis is also in line with previous findings [12,18]. Additionally, we hypothesized that this phenomenon will be more pronounced for individuals with intuitive and spontaneous decision-making styles compared to those with a rational style, since the rational decision-makers are more engaged with System 2 and may be more likely to analyze both scenarios in terms of cost versus benefit and to realize that if the gift remains constant, spending less on it should not affect its value negatively.

Buying a gift in a scenario that does not mention being abroad raises the possibility that the recipient will reveal the gift’s price (e.g., by seeing it in a store or by trying to replace it), and then the reasons not to save on the gift become even stronger. Therefore, in this version of the experiment, we expect the difference between the time valuation in the two buying purposes (buying a gift versus buying for oneself) to be higher than in the case of buying abroad.

Another effect of being abroad is that one’s time is likely to be more valuable compared to its value when not traveling abroad. Often people are abroad for vacation, which means they have limited time, they pay for flights, hotels, and transportation, they want to make good use of their vacation time, etc. We hypothesize that people who are characterized by a more rational decision-making style [2] will reflect this understanding that time abroad is more valuable more than others with a different decision-making style.

## 3. Experiment 1: Discount Required for a Defined Effort Abroad

### 3.1. Materials and Methods

To examine the influence of the buying purpose on the valuation of time, we used an online recruitment platform (Prolific) and recruited 200 British subjects who were paid for their participation in the experiment. We distributed a questionnaire (using Qualtrics) that asked about three buying scenarios. It then included 15 decision-making style questions [2] to evaluate rational, intuitive, and spontaneous decision-making. Finally, it included some demographic questions. Appendix A includes the 15 decision-making style questions and the demographic questions. The experiment was conducted between-subjects, and subjects were divided randomly between two treatments, which differed only by the buying purpose described in the buying scenarios. One treatment asked about buying a gift for someone else, and the second treatment asked about buying for self-use. To examine the robustness of our findings, we used three scenarios that differ in the reason a gift is being purchased and in the type of good being purchased. We wanted the scenarios to be realistic, so we used different goods that are reasonable to purchase both for oneself and as a gift, and that are reasonable to also purchase abroad. In particular, subjects were asked the following questions (the second treatment in brackets):

*1. You travel abroad and find a book that you want to buy for yourself for £28 [You travel abroad and find a book that you want to buy as a gift for the birthday of a close friend for £28]. You got to know that the book is sold for a lower price at a store located a twenty-minute drive away. What is the maximum price at the remote store that requires driving, for which you will prefer to buy there and not at the store you currently visit? £___________*

*2. You travel abroad and see a nice set of silverware that you want to buy for yourself, sold for £89 [You travel abroad and see a nice set of silverware sold for £89. You are planning to visit next month a good friend who lives outside your town. He invited you to stay at his apartment for a week and given his generous hospitality you want to buy him the silverware set as a gift]. You search on your smartphone and find that the same silverware set is offered at another store for a cheaper price, but this other store requires a fifteen-minute walk. What is the maximum price at the remote store that requires walking, for which you will prefer to buy there and not at the store you currently visit? £___________*

*3. You travel abroad and want to buy a chocolate box for yourself [You travel abroad and want to buy a chocolate box for your cousin, who invited you to a holiday dinner next month]. You see the chocolate box at the supermarket you visit for £43, and your online search shows it also at another store that requires taking a bus for ten minutes. What is the maximum price at the remote store that requires taking the bus, for which you will prefer to buy there and not at the supermarket you currently visit? £___________*

### 3.2. Results and Discussion

Each of the 200 subjects answered about three buying scenarios for a total of 600 potential decisions. In two cases, we did not receive a response at all, and in two cases, non-usable responses were received, so we ended up with 596 observations (in one case, the subject wrote that he does not drive; in the second, the response indicated zero discount required to make the effort of traveling to the remote store). We computed for each answer the minimal discount the subject required in order to buy in the remote store. This minimal discount is computed as the difference between the response provided by the subject and the price mentioned in the question. For example, in the first question the price mentioned was GBP 28, and subjects were asked “What is the maximum price at the remote store that requires driving, for which you will prefer to buy there and not at the store you currently visit?” A subject who answers GBP 22 implies that the minimal discount for which they are willing to make the twenty-minute drive effort is GBP 6 (=28−22). Table 1 presents the variables we use in the analysis.

To analyze the effect of the buying purpose, we ran a regression with the dependent variable of the minimal discount required and the explanatory dummy variable Yourself (equal to 1 in the treatment of buying for oneself and 0 when buying a gift for someone else), in addition to dummy variables for the different scenarios (the book scenario being the omitted benchmark). Table 2 presents the results:

The regression suggests that there is no statistically significant effect of buying purpose. Subjects who were told that the product is a gift for someone else did not ask for a statistically significant different discount compared to those who were told that the product is for themselves. Our hypothesis that people derive some utility from buying a more expensive gift for others and therefore will make less effort to save when buying them a gift is not supported by the data. Not only is there no significant effect, but also the positive sign of the variable Yourself goes in the opposite direction to the hypothesized effect: people asked for a greater discount on average when buying for themselves. To conclude, we found no influence of purchase purpose on the trade-off between time and money.

The results may have two possible explanations. The first is the influence of buying abroad. We hypothesized that the buyer obtains some utility from a more expensive gift (or suffers some disutility from buying a cheaper gift). This is likely to be stronger when the recipient has more chances to discover the gift’s price. Possibly, buying abroad, which means that the recipient is very unlikely to find the gift’s price, erodes completely or to a large extent the utility of buying a more expensive gift, and therefore people are no longer making more effort to save on buying for themselves compared to buying a gift. To address this possibility, we will report below an additional experiment, in which the scenarios will not be described as taking place abroad, and compare the results to those of Experiment 1.

The second possible reason for the lack of a clear effect is that two contradicting effects exist, both due to the same cause that gifts are measured, at least partially, by the price paid for them. On the one hand, as mentioned earlier, when buying a gift for someone else, a person may ask for a greater discount to travel to a remote store (compared to buying for oneself) because the utility from saving a certain amount is lower. On the other hand, the buyer does not feel comfortable with buying a “cheap” gift and wants to avoid it, so they benefit less from saving on the gift. Consequently, they do not ask for a large discount in return for the effort of traveling to a different store. In order to avoid this situation that the same phenomenon (utility from buying a more expensive gift) results in two opposite effects and makes it hard to gain insights, we conducted an additional experiment, described in the next section.

## 4. Experiment 2: Effort Acceptable for a Defined Saving Abroad

### 4.1. Materials and Methods

In Experiment 2, we created three scenarios similar to the ones in Experiment 1, but now we asked the subjects to indicate the maximal effort (in terms of time spent) they are willing to exert to save a predefined amount of money on the product they purchase. This keeps the trade-off we are interested in of time versus money but eliminates the problem mentioned in the previous section of a potential opposite effect (of unwillingness to buy a cheap gift). Here, if people are less willing to make the effort to save when buying a gift (compared to buying for themselves), they should indicate this by specifying a shorter amount of time they are willing to invest in order to save when they buy a gift for others. In particular, we used the following three scenarios (the second treatment in brackets):

*1. You travel abroad and find a book that you want to buy for yourself for £28 [You travel abroad and find a book that you want to buy as a gift for the birthday of a close friend for £28]. You got to know that the book is sold for £21 at a remote store. What is the maximum driving time in minutes that you are willing to drive to that remote store, for which you will prefer to buy there and not at the store you are currently visit? __________ driving minutes*

*2. You travel abroad and see a nice set of silverware that you want to buy for yourself, sold for £89 [You travel abroad and see a nice set of silverware sold for £89. You are planning to visit next month a good friend who lives outside your town. He invited you to stay at his apartment for a week and given his generous hospitality you want to buy him the silverware set as a gift]. You search on your smartphone and find that the same silverware set is offered at another store for £73, but this other store requires walking. What is the maximum walking time in minutes that you are willing to walk to that remote store, for which you will prefer to buy there and not at the store you currently visit? __________walking minutes*

*3. You travel abroad and want to buy a chocolate box for yourself [You travel abroad and want to buy a chocolate box for your cousin, who invited you to a holiday dinner next month]. You see the chocolate box at the supermarket you visit for £43, and your online search shows it also sells at another store for £32, but that store requires taking a bus. What is the maximum bus riding time in minutes that you are willing to ride to that remote store, for which you will prefer to buy there and not at the supermarket you currently visit? __________riding minutes*

We, again, used the Prolific platform for recruitment, a Qualtrics questionnaire, and recruited 200 British subjects who were paid for their participation in the experiment. The scenarios were again followed by the 15 decision-making style questions and some demographic questions.

### 4.2. Results and Discussion

Each of the 200 subjects answered about three buying scenarios for a total of 600 decisions. To analyze the effect of the buying purpose, we ran regression (1) with the dependent variable of Time, the maximal acceptable effort in minutes (in order to save the predefined amount of money), and the explanatory dummy variable Yourself, in addition to the dummy variables for the different scenarios (the book scenario being the omitted benchmark). In 48 observations, the variable Time is equal to zero, a response that does not make much sense. We, therefore, also analyze a regression where we omit these 48 observations. Table 3 presents the results.

As mentioned above, we hypothesized that people are willing to invest less time in obtaining a certain discount when buying a gift for others because the gift buyer’s utility from the discount is somewhat offset by the disutility of giving a cheaper present. However, the results of the regression (1) show an effect (*p* = 0.035) in the opposite direction. The willingness to invest time and go to the cheaper store for subjects who were told that the product is for themselves was reduced by about 2.53 min. A similar result is obtained also when we limit attention to observations with strictly positive values of Time, reported in regression (2).

Because, in this experiment, we obtain statistically significant results about the variable Yourself (albeit in an unexpected direction), it may be interesting to analyze whether the decision-making styles interact with Yourself. Recall that we have three scores (intuitive, spontaneous, and rational) based on the answers to the 15 decision-making style questions. Each score is between 1 and 5 and is based on answers to five unique questions, so it is possible that a subject will be high in intuitive or spontaneous scores and also in the rational score, for example. To understand how the different decision-making styles are related, Table 4 presents the correlation between the three scores.

As Table 4 shows, there is a negative correlation between the rational and the other two styles and a positive correlation between the spontaneous and the intuitive styles.

Regression (3) adds the three decision-making scores and their interaction with Yourself, and regression (4) uses the same variables but limits the analysis to observations with strictly positive value of Time. The results in both regressions show that the three decision-making scores and their three interactions are not statistically significant.

To summarize, we found that people are willing to invest less time in obtaining a certain discount when buying for themselves, contrary to our prediction. We conjecture that this is related to the purchase being made abroad, reducing the likelihood that the gift recipient will reveal the purchase price, and also implying that they cannot replace the gift, which in turn decreases the desire to buy a gift at a high price. Additionally, we hypothesized that the influence of whether the purchase is for oneself or for someone else would be stronger for intuitive and spontaneous decision-makers compared to rational decision-makers. However, this hypothesis was based on our main hypothesis about buying for oneself versus buying a gift being supported, and thus this is no longer relevant once the main hypothesis is not supported by the data.

## 5. Experiment 3: Discount Required for a Defined Effort Not Abroad

As we mentioned earlier, being abroad affects the ability of the gift recipient to find out the gift’s price; it implies not being able to replace the gift, and it affects the value of the buyer’s time. To address some issues we discussed earlier, and, in particular, find whether buying a gift when not being abroad results in less willingness to make an effort to save, we therefore repeated Experiment 1 but without the scenarios being described as taking place abroad. This will also allow us to analyze whether people express a higher valuation of their time when being abroad, as seems reasonable.

### 5.1. Materials and Methods

We conducted the same experiment as Experiment 1 with the only difference being the omission of the words about traveling abroad. We again used the Prolific platform for recruitment, a Qualtrics questionnaire, and recruited 200 British subjects who were paid for their participation in the experiment. The scenarios were again followed by the 15 decision-making style questions and some demographic questions. The three scenarios were as follows (the second treatment in brackets):

*1. You find a book that you want to buy for yourself for £28 [You find a book that you want to buy as a gift for the birthday of a close friend for £28]. You got to know that the book is sold for a lower price at a store located a twenty-minute drive away. What is the maximum price at the remote store that requires driving, for which you will prefer to buy there and not at the store you currently visit? £___________*

*2. You see a nice set of silverware that you want to buy for yourself, sold for £89 [You are planning to visit next month a good friend who lives outside your town. He invited you to stay at his apartment for a week and given his generous hospitality you want to buy him the silverware set as a gift]. You search on your smartphone and find that the same silverware set is offered at another store for a cheaper price, but this other store requires a fifteen-minute walk. What is the maximum price at the remote store that requires walking, for which you will prefer to buy there and not at the store you currently visit? £___________*

*3. You want to buy a chocolate box for yourself [ your cousin, who invited you to a holiday dinner next month]. You see the chocolate box at the supermarket you visit for £43, and your online search shows it also at another store that requires taking a bus for ten minutes. What is the maximum price at the remote store that requires taking the bus, for which you will prefer to buy there and not at the supermarket you currently visit? £___________*

### 5.2. Results and Discussion

Each of the 200 subjects answered about three buying scenarios for a total of 600 potential decisions. Of these 600 decisions, 6 did not ask for a strictly positive discount in order to make the effort of traveling to the remote store, which suggests a typo or not answering seriously, and we omit these six observations. Table 5 presents the results of several regressions.

As we can see from regression (1), when we asked about the discount requested without writing that the travel is abroad, the coefficient of Yourself became negative (participants were willing to ask for a smaller discount when buying for themselves), in line with our hypothesis and as opposed to the positive coefficient in Experiment 1. However, these coefficients in both experiments are not statistically significant.

To analyze more carefully the effect of being abroad, we combined the data of traveling Abroad (Experiment 1) with not abroad (Experiment 3). The dummy variable abroad is equal to 1 in Experiment 1 and to 0 in Experiment 3. Regression (2) shows that the coefficient of Abroad is positive and statistically significant. This confirms our hypothesis that people value their time abroad as more valuable, which makes sense given that being abroad often means being on vacation, paying for flights and hotels, etc.

Next, we also add the interaction between abroad and yourself, written as AbroadXYourself (similarly, other variables that include X are the interaction terms between the variables before and after X). Regression (3) shows that the coefficient of the interaction AbroadXYourself is also positive. Although this interaction is not statistically significant (*p* = 0.058) in regression (3), it is statistically significant in some other regressions that will be discussed below. Recall that yourself has a negative coefficient, which may be due to the possibility that the recipient will replace the gift or at least find its price. Because being abroad implies that the gift will not be replaced and its price is unlikely to be revealed to the recipient, this erodes the potential desire of the buyer to purchase a more expensive gift and implies that the buyer becomes more willing to make an effort to save on a gift, i.e., they require a lower discount on a gift (compared to buying for themselves), or equivalently, a higher discount on buying for themselves (compared to buying a gift).

In regression (4), we add the analysis of the decision-making styles. In regression (4), we find that Rational has a negative coefficient, meaning that subjects who tend more to rational decision-making asked for lower discounts (reflected a lower valuation of their time). Generally, the discounts people require in the experiment reflect a much higher valuation of their time compared to how much they earn from completing tasks on Prolific, so it makes sense that those who think more rationally than others also reflect a lower valuation of time (which is therefore closer to reality) in the scenarios. The three scenarios mention a store that requires spending 20, 15, or 10 min to get to it, or 15 min on average. The average discount required by subjects in Experiments 1 and 3 is slightly above GBP 11, implying time valuation a little over GBP 44 per hour. On the other hand, subjects often complete tasks on Prolific for less than GBP 10 per hour, and presumably this reflects their correct time valuation. We also find again a positive coefficient for the interaction of Abroad and Yourself that we discussed above, but here it is also statistically significant (*p* = 0.036).

Regression (5) adds also interactions of decision-making styles with Yourself and Abroad. In addition to a negative coefficient of Rational and positive coefficient for the interaction of Abroad and Yourself as in regression (4), we see here also a positive interaction of Rational and Abroad (*p* = 0.010). This suggests that subjects who tend more to rational thinking reflect more the consideration that time when being abroad is more valuable than when not being abroad.

Finally, we wanted to run another robustness check by using a somewhat different dependent variable. Instead of considering the total discount required, we compute the discount required per minute by dividing the total discount in each response by the number of minutes that it takes to reach the cheaper store in that scenario. Regression (6) uses this alternative dependent variable. Because the dependent variable is different, the level of the coefficients is different. The qualitative results, however, remain unchanged: variables that were previously statistically significant remain statistically significant and in the same direction.

## 6. Conclusions

We study the trade-off between time and money in the decision of whether to travel to a cheaper store to save money on a product we want to buy. We consider the case of buying something for ourselves versus buying it as a gift for someone else. The literature reports differences in how we treat expenditures on gifts compared to other expenditures. Based on this, we hypothesized that when purchasing a gift, one is less enthusiastic about saving money because they have some utility from buying a more expensive gift; they do not want to buy a gift that is too cheap; and because the recipient may reveal the gift’s price or even try to replace it, both giving an incentive to buy a more expensive gift. Consequently, we hypothesized that people will be less willing to spend time traveling to a cheaper store to save money when they buy a gift (compared to when buying for themselves). In a set of experiments, we fail to find support for this hypothesis. In particular, and contrary to our hypothesis, we found that when purchasing abroad, individuals demand a higher discount when buying for themselves than when buying a gift (though this is not statistically significant) and are willing to invest less time in obtaining a predefined discount (this is statistically significant).

In addition, we hypothesized that the trade-off between time and money would vary according to the decision-maker’s style. Specifically, we expected that intuitive and spontaneous decision-makers would exhibit a more pronounced difference in this trade-off compared to rational decision-makers. This hypothesis was based on the conjecture that some subjects will exhibit behavior that may be claimed to be not fully rational by making less effort to save on a gift compared to purchasing for themselves. Then, it makes sense to expect that rational decision-makers will exhibit this non-rational behavior less than others. But once the general population does not exhibit the hypothesized main effect, there is no longer a reason to expect that rational decision-makers will behave differently from others. From the perspective of the dual-system approach, if System 1 is not biased in this context, operating System 2 cannot overcome a bias and change behavior.

We also explore the impact of telling subjects that the purchase is made abroad (versus not mentioning being abroad). This is hypothesized to increase the valuation of the buyer’s time, as being abroad often implies being on vacation, having to pay for hotels and flights, etc. The data support this hypothesis. We also hypothesize that the interaction of being abroad and buying a gift will be negative, because when we buy abroad, the gift recipient is unlikely to find out the gift’s price and is unable to replace it, both eroding the desire to spend more on the gift. The data also support this hypothesis.

The theoretical implications of our research include a deeper understanding of the trade-off between time and money. This topic is significant because people make decisions related to this trade-off on a daily basis. The decision of how many hours to work is one example that illustrates this trade-off. Other examples include the decision in which store to buy, when some stores are closer (and therefore save time) but are more expensive (as is often the case between city center stores versus stores in the outskirts of the city). The decision of how much time to invest in searching for prices in additional shops is also an example of a decision that involves the trade-off between time and money. We enrich the existing literature on this topic by studying another possible influence—whether the purchase is for oneself or for others. The research also has practical implications, for example, for firms that should decide where to locate their stores in general, and, in particular, taking into account whether the store sells goods that are often being purchased as gifts or not.

We ask the subjects 15 questions about their decision-making style [2], which yield scores about their tendency to exercise rational, intuitive, and spontaneous decision-making, and analyze their decisions also with respect to these scores. We find that subjects who score higher in terms of rational decision-making reflect a lower time value in their decisions. A lower time value is indeed more “rational” in the sense that the answers to the scenarios reflect, on average, a time value of over GBP 44 per hour, whereas the subjects are people who solve Prolific tasks, which often pay less than GBP 10 per hour. In addition, subjects with a higher score in terms of rational decision-making reflect more the consideration that time abroad is more valuable (the interaction between rational and abroad is positive). This again makes sense and suggests that the questions about decision-making style are correlated with decision-making in scenarios of realistic purchase decisions. In terms of the dual-system approach, it seems that people who operate System 2 more and therefore are also higher in the rational decision-making score are better calibrated about the value of their time and make more informed decisions about the trade-off between time and money. Possibly the decisions that first come to mind (i.e., the outcome of System 1) tend to over-estimate one’s value of time and not to express sufficiently that time abroad is more valuable. Those who operate System 2 more significantly (i.e., rational decision-makers), however, are better able to overcome these initial mistakes. Therefore, they reflect time valuation that is closer to the real time value and take into consideration better the circumstances such as buying abroad when time value is higher.

These latter results have significant theoretical and practical implications as they emphasize the importance of considering heterogeneity in the subject pool when conducting experiments. If we only analyze the average behavior (for example, in a certain experimental treatment), we fail to understand how personal characteristics affect behavior. In some cases, we may not even observe an effect of a certain factor because it affects different people in opposite directions. But if the manner in which a factor affects behavior is systematic, meaning that we can measure a certain aspect of personal characteristics, and this aspect helps to predict behavior, we gain a better understanding of behavior in this context. This can be true about demographics that are objective and easier to collect, such as age, gender, and education. But as we show here, it is also true about decision-making styles. This suggests that both academic studies and non-academic studies (e.g., by firms that want to gain insights into consumer preferences and behavior, or governmental agencies that want to understand citizens’ behavior) can benefit from collecting data about the personal characteristics of people, including about their decision-making styles. This will allow them to better understand how different people may react differently to marketing ads, governmental policies, etc.

Turning to the limitations of this study, one limitation is the use of participants through an online platform. This may cause a lack of engagement from participants, which could lead to less accurate or thoughtful responses. Participation in such online experiments is limited to people who register for the relevant platforms such as Amazon M-Turk or, in our case, Prolific. They are not necessarily representative of the general population, although we do not have a reason to believe that their behavior in terms of trading-off time and money when buying for themselves versus buying a gift is systematically different from that of the general population.

The samples in the various experiments were of British participants (so that we could use GBP as a currency that every subject is used to). Possibly in cultures where social norms about gifts are substantially different, behavior when buying gifts may also differ and produce different results. However, not every aspect of gift giving that differs between cultures is relevant to the topic we studied. We focused on the willingness of the buyer to make an effort to save on the gift (without sacrificing its quality, since they buy the exact same gift only in a cheaper store). So, if in one culture wine is a common gift and in another culture chocolate or flowers are common gifts, but there is no significant difference in how much people in the different cultures try to save when buying a gift, these cultural differences will not be relevant to our study. What matters for our results is how much the actual cost of the gift is important to the giver. If Western cultures are similar in this regard, the findings may be applicable to other Western countries as well. An avenue that is more likely to obtain different results may be to consider not other Western countries but rather countries with a completely different culture, for example, East Asian or Muslim countries.

Finally, participants were paid a fixed amount and not compensation that depends on their decisions, similar to many other studies involving hypothetical scenarios. Therefore, they did not have financial incentives to provide truthful responses. However, this methodology of hypothetical scenarios is very common in psychology. The subjects also did not have a particular reason to misrepresent their true preferences. In addition, it is difficult to create real situations with real purchases where one must buy a certain good for themselves, can buy the good in two different places where one is closer but more expensive, and others must buy the exact same good at the same two stores but as a gift they want to give to someone else.

In terms of ideas for future research, one idea, implied by the limitation mentioned above, is to run similar experiments in other cultures and, in particular, ones that substantially differ in terms of their social norms about gifts. Another idea is to examine how the results may be affected by emphasizing in the scenario the possibility that the gift recipient may discover its price or may want to replace it. A third idea is to examine not the difference between buying for ourselves versus buying a gift as we did but rather the difference between buying a gift for someone close (e.g., our parents, brother, or a close friend) versus buying a gift for someone less close (e.g., a remote relative or friend, or a colleague who is not close). Finally, it could be interesting to explore the impact of the reason a gift is purchased on the trade-off between time and money, for example, between a gift we buy because we are invited to dinner, a gift for a wedding, a gift for a birthday, etc.

## Figures and Tables

**Table 1:** Summary of variables.

Variable	Meaning	
Discount	Minimal discount required to make the effort of going to the remote store	
Yourself	Equals 1 when buying for oneself	Equals 0 when buying a gift
q silver	Equals 1 for silverware question	Equals 0 for the other questions
q chocolate	Equals 1 for chocolate question	Equals 0 for the other questions
Rational (or Rati in interaction terms)	The average response to the five rational decision-making style questions (between 1 and 5).	
Intuitive (or Intu in interaction terms)	The average response to the five intuitive decision-making style questions (between 1 and 5).	
Spontaneous (or Spon in interaction terms)	The average response to the five spontaneous decision-making style questions (between 1 and 5).	
Time	Time in minutes required by the subject in Experiment 2	
Abroad	Equals 1 when buying scenario is mentioned to occur abroad	Equals 0 when it is not mentioned to occur abroad

**Table 2:** Experiment 1—regression results.

Independent Variables	Dependent Variable: Discount
Yourself	0.685 (*p* = 0.266)
q silver	3.874 *** (*p* = 0.000)
q chocolate	2.655 *** (*p* = 0.000)
constant	9.410 *** (*p* = 0.000)
N	596
*Adj R* ^2^	0.042
*F*	9.65
*Prob* > *F*	0.000

The scenario about book purchase serves as the benchmark. *p*-values are reported in parentheses. Levels of statistical significance are denoted by asterisks: *** *p* < 0.001.

**Table 3:** Experiment 2—regression results.

Dependent Variable Independent Variables	(1) Time	(2) Time > 0	(3) Time	(4) Time > 0
Yourself	−2.532 * (*p* = 0.035)	−2.501 * (*p* = 0.042)	−14.412 (*p* = 0.246)	−8.435 (*p* = 0.510)
q silver	12.500 *** (*p* = 0.000)	12.075 *** (*p* = 0.000)	12.500 *** (*p* = 0.000)	12.048 *** (*p* = 0.000)
q chocolate	−0.320 (*p* = 0.828)	3.047 * (*p* = 0.049)	−0.320 (*p* = 0.828)	3.032 * (*p* = 0.050)
Rational			−1.450 (*p* = 0.366)	−1.888 (*p* = 0.257)
Intuitive			0.235 (*p* = 0.876)	0.761 (*p* = 0.618)
Spontaneous			−2.028 (*p* = 0.158)	−2.233 (*p* = 0.129)
RatiXYourself			2.468 (*p* = 0.256)	1.607 (*p* = 0.476)
IntuXYourself			−0.868 (*p* = 0.708)	−1.107 (*p* = 0.636)
SponXYourself			2.048 (*p* = 0.312)	1.399 (*p* = 0.497)
constant	17.304 *** (*p* = 0.000)	17.850 *** (*p* = 0.000)	27.585 ** (*p* = 0.004)	28.449 ** (*p* = 0.004)
N	600	552	600	552
*Adj R* ^2^	0.144	0.120	0.139	0.116
*F*	34.45	25.99	11.76	9.01
*Prob* > *F*	0.000	0.000	0.000	0.000

The scenario about book purchase serves as the benchmark. *p*-values are reported in parentheses. Levels of statistical significance are denoted by asterisks: * *p* < 0.05, ** *p* < 0.01, *** *p* < 0.001.

**Table 4:** The correlation between the decision-making style scores.

	Rational	Intuitive	Spontaneous
Rational	1.000		
Intuitive	−0.058 * (*p* = 0.014)	1.000	
Spontaneous	−0.474 *** (*p* = 0.000)	0.486 *** (*p* = 0.000)	1.000

*p*-values are reported in parentheses. Levels of statistical significance are denoted by asterisks: * *p* < 0.05, *** *p* < 0.001.

**Table 5:** Experiment 3 alone and combined with Experiment 1.

	(1)	(2)	(3)	(4)	(5)	(6)
Dependent Variable	Discount	Discount	Discount	Discount	Discount	Discount per Minute
Indep. Variables						
Yourself	−0.824 (*p* = 0.093)	−0.069 (*p* = 0.861)	−0.822 (*p* = 0.142)	−0.874 (*p* = 0.12)	0.901 (*p* = 0.811)	0.037 (*p* = 0.898)
q silver	0.418 (*p* = 0.487)	2.156 *** (*p* = 0.000)	2.155 *** (*p* = 0.000)	2.151 *** (*p* = 0.000)	2.151 *** (*p* = 0.000)	0.303 *** (*p* = 0.000)
q chocolate	2.486 *** (*p* = 0.000)	2.575 *** (*p* = 0.000)	2.572 *** (*p* = 0.000)	2.570 *** (*p* = 0.000)	2.572 *** (*p* = 0.000)	0.735 *** (*p* = 0.000)
Rational				−0.721 * (*p* = 0.043)	−1.584 * (*p* = 0.012)	−0.129 ** (*p* = 0.008)
Intuitive				0.443 (*p* = 0.225)	0.726 (*p* = 0.276)	0.072 (*p* = 0.156)
Spontaneous				−0.340 (*p* = 0.32)	−0.283 (*p* = 0.628)	−0.041 (*p* = 0.360)
RatiXYourself					−0.034 (*p* = 0.962)	0.009 (*p* = 0.872)
IntuXYourself					0.362 (*p* = 0.621)	0.021 (*p* = 0.712)
SponXYourself					−1.136 (=0.097)	−0.083 (*p* = 0.112)
RatiXAbroad					1.844 ** (*p* = 0.010)	0.145 ** (*p* = 0.008)
IntuXAbroad					−1.073 (*p* = 0.145)	−0.101 (*p* = 0.072)
SponXAbroad					0.995 (*p* = 0.146)	0.097 (*p* = 0.062)
Abroad		1.609 *** (*p* = 0.000)	0.854 (*p* = 0.129)	0.772 (*p* = 0.171)	−5.331 (*p* = 0.163)	−0.423 (*p* = 0.146)
AbroadXYourself			1.503 (*p* = 0.058)	1.664 * (*p* = 0.036)	1.655 * (*p* = 0.039)	0.123 * (*p* = 0.044)
constant	9.768 *** (*p* = 0.000)	8.786 *** (*p* = 0.000)	9.163 *** (*p* = 0.000)	11.368 *** (*p* = 0.000)	13.631 *** (*p* = 0.000)	0.818 ** (*p* = 0.002)
N	594	1190	1190	1190	1190	1190
*adj R^2^*	0.032	0.036	0.039	0.040	0.044	0.2629
*F*	7.51	12.23	10.53	7.19	4.92	31.31
*Prob* > *F*	0.000	0.000	0.000	0.000	0.000	0.000

The dependent variable is the discount required. *p*-values are reported in parentheses. Levels of statistical significance are denoted by asterisks: * *p* < 0.05, ** *p* < 0.01, *** *p* < 0.001.

## Data Availability

The raw data supporting the conclusions of this article will be made available by the authors on request.

## Appendix A. The 15 Decision-Making Style Questions and the Demographic Questions

We adopted the questions related to three decision-making styles: rational, intuitive, and spontaneous, as commonly used in the literature [2]. In the questionnaire that our participants answered, we mixed the order of the 15 questions without telling our participants to which decision-making style each question belongs. Each question was rated on a scale of 1 (strongly disagree) to 5 (strongly agree).

The five rational decision-making style questions are as follows:

I double-check my information sources to be sure I have the right facts before making decisions.

I make decisions in a logical and systematic way.

My decision making requires careful thought.

When making a decision, I consider various options in terms of a specific goal.

I explore all of my options before making a decision.

.

The five intuitive decision-making style questions are as follows:

When making decisions, I rely upon my instincts.

When I make decisions, I tend to rely on my intuition.

I generally make decisions that feel right to me.

When I make a decision, it is more important for me to feel the decision is right than to have a rational reason for it.

When I make a decision, I trust my inner feelings and reactions.

.

The five spontaneous decision-making style questions are as follows:

I generally make snap decisions.

I often make decisions on the spur of the moment.

I make quick decisions.

I often make impulsive decisions.

When making decisions, I do what seems natural at the moment.

.

Additional questions:

Gender: male/female

Age: ______________

Years of schooling so far, counting everything from elementary school to university: _______

Academic field of undergraduate studies: ______________

How many university-level courses in economics did you take? ______________

If you have any comments, please write them down here: ______________
